# Hepatitis C virus infection in blood donors in Santa Catarina, Brazil, 2010–2020

**DOI:** 10.1111/tme.70004

**Published:** 2025-07-24

**Authors:** Fabiana Schuelter‐Trevisol, Gabriel Tadeu Ossani, Gabriel Oscar Cremona Parma, Daisson José Trevisol

**Affiliations:** ^1^ Programa de Pós‐Graduação em Ciências da Saúde Universidade do Sul de Santa Catarina Brazil; ^2^ Curso de Medicina Universidade do Sul de Santa Catarina Brazil

**Keywords:** anti‐hepatitis C antibodies, blood donors, donor selection, epidemiology, hepatitis C

## Abstract

**Introduction:**

Hepatitis C is a silent disease characterised by a persistent inflammatory process in the liver. Since blood is the main route of transmission, the objective of this study was to estimate the positivity rate of hepatitis C virus (HCV) infection in blood donations from the Public Blood Network of the State of Santa Catarina and to analyse the temporal trend and spatial distribution of cases between 2010 and 2020.

**Methods:**

This historical cohort study included blood donors who tested positive for HCV and donated blood at the service between January 1, 2010, and December 31, 2020.

**Results:**

Out of a total of 1 316 605 blood donations, 782 new samples tested positive for HCV, corresponding to an overall positivity rate of 59.4 per 100 000 donations. The majority of HCV‐positive donors were adults, male, of white skin colour, married, and had at least a secondary education. Most HCV‐positive cases were found in the South and Coastal Regions of Santa Catarina, although municipalities with high positivity rates were observed across all macro‐regions of the state.

**Conclusions:**

The HCV positivity rate in blood donations from the Public Blood Network of the State of Santa Catarina demonstrated a downward trend over time, while maintaining sociodemographic characteristics similar to those reported in other regional studies. Furthermore, although HCV cases were reported in all macro‐regions, the highest positivity rates occurred in the South, Alto Vale do Itajaí, Meio Oeste, Serra Catarinense, and Grande Oeste regions.

## 1 INTRODUCTION

Blood transfusion is a widely used therapeutic procedure for various clinical conditions and diseases. However, transfused blood can pose risks to patients if contaminated with high‐risk infectious diseases, such as viral hepatitis.^1^ Approximately 70 million people worldwide are infected with the hepatitis C virus (HCV), which is 10 times more infectious and transmissible than the human immunodeficiency virus (HIV).^2^

HCV infection is primarily transmitted parenterally through contact with contaminated blood, including the use of blood and blood products.^3^ However, diagnosis is usually made during routine serological testing or blood donation, through the detection of anti‐HCV antibodies and nucleic acid amplification testing (NAT).^4^, ^5^ This highlights the critical role of blood centres in ensuring the provision of safe blood components and maintaining a strong commitment to the safety of both donors and recipients.^6^ It is estimated that most hepatitis C cases are asymptomatic, with approximately 50% being diagnosed through routine testing.^4^

HCV infection is characterised by a subclinical progression and a persistent inflammatory process in the liver.^7^ Complications of HCV infection include cirrhosis, hepatocellular carcinoma, liver transplantation, and death. Moreover, the disease burden and the associated healthcare and economic costs to public institutions may increase as the infected population progresses to severe liver disease.^8^, ^9^

The prevalence of HCV exhibits significant variability depending on geographic regions and sociodemographic characteristics, a pattern partially attributed to the individualised nature of its transmission. Furthermore, the ongoing challenge of developing an effective primary prevention strategy remains a major public health concern. Despite extensive research in recent years on anti‐HCV vaccines targeting various viral antigens and employing alternative delivery systems, success has been limited due to the virus's high genetic variability and adaptive capacity.^10^

Recent advancements in antiviral therapy have demonstrated high efficacy, particularly when treatment is initiated at an early stage of infection.^11^, ^12^ In response to the global burden of HCV, the World Health Organization has set an ambitious goal to eradicate hepatitis C by 2030, aiming for a 90% reduction in new infections and a 65% reduction in HCV‐related mortality. Achieving these targets necessitates the implementation of comprehensive prevention, diagnosis, and treatment strategies, alongside robust healthcare support systems. However, it is estimated that 80% of individuals living with hepatitis lack adequate access to essential healthcare services.^13^

In Brazil, an estimated 0.7% of the population is infected with HCV, corresponding to approximately 700 000 people. However, a significant portion remain unaware of their serological status. Additionally, the serological window for HCV is relatively long (around 70 days), but third‐generation serological tests used in blood banks can detect prior contact with the virus.^14^

Since 2015, the introduction of direct‐acting antivirals has led to a significant reduction in hepatitis C‐related mortality and a 57% decrease in new HCV infections. Although treatment can achieve cure rates of up to 95%, thereby reducing mortality and interrupting the transmission chain, it is important to note that individuals with prior exposure to HCV remain ineligible for blood or blood product donation.^14^, ^15^

Given the severity of hepatitis C and the critical role of preventive measures and early diagnosis, this study aims to estimate the positivity rate of HCV infection in blood donations within the Public Blood Network of the State of Santa Catarina, Brazil. Additionally, it seeks to analyse the temporal trend and spatial distribution of cases over the period from 2010 to 2020.

## 2 METHODS

This study was approved by the Research Ethics Committee of the *Universidade do Sul de Santa Catarina* (UNISUL, University of Southern Santa Catarina), under Opinion No. 5251067, on February 18, 2022, and by the Research Ethics Committee of the *Centro de Hematologia e Hemoterapia de Santa Catarina* (HEMOSC, Center for Hematology and Hemotherapy of Santa Catarina), under Opinion No. 5344297, on April 11, 2022.

This was an observational epidemiological study with a historical cohort design. All blood samples with confirmed positive result for hepatitis C from the Public Blood Network of the State of Santa Catarina between January 1, 2010, and December 31, 2020, were analysed. The sample was derived from the total blood donations in the State of Santa Catarina during this period that tested positive for HCV.

Candidates deemed eligible for blood donation after screening through interviews and physical examinations, who donated blood at the Public Blood Network during the selected period and tested positive for anti‐HCV (Alinity S and Architect I2000, Abbott®) and/or NAT (Kit NAT HIV/HCV/HBV, Bio‐Manguinhos®), were included in the study. As per the service's protocol, all donated blood samples are tested, regardless of donor type. In the period of the study, the implementation of NAT for HCV began in 2013.

The Public Blood Network of the State of Santa Catarina has its coordinating center located in the capital, Florianópolis. Additionally, there are six regional centers in the cities of Criciúma, Lages, Joaçaba, Chapecó, Joinville, and Blumenau (See Figure S1—map). The state also has eight transfusion agencies and two blood collection units (See map—Coverage Map of the Blood Network in Santa Catarina). To provide a clearer understanding of the service, the following terms are defined.

- *First‐time donor*: An individual donating blood for the first time at that blood service.
- *Repeat donor*: A donor who makes two or more donations within a 12‐month period.
- *Sporadic donor*: A donor who donates again after an interval of more than 12 months since their last donation.
- *Voluntary donation*: A donation made by individuals motivated to maintain the blood bank's stock as an altruistic act, without identifying a specific recipient.
- *Replacement donation*: A voluntary and altruistic donation, typically associated with a hospitalised patient, made to replenish the blood used or to be used for that patient.

Testing for hepatitis C in blood donors follows current Brazilian legislation.^16^ All donated blood samples undergo both serological testing and nucleic acid testing (NAT), as these tests are complementary.

- If both tests are negative, the blood unit is approved for transfusion.
- All samples are tested in triplicate in serological assays.
- NAT testing is performed on pools of six samples. If NAT results do not match the serological findings, the sample is tested individually.
- If both tests are positive, or if only the NAT test is positive, the blood unit is discarded and the donor is considered infected with hepatitis C.
- If only the serological test is positive, the sample is retested. In such cases, the donor is contacted for a new sample collection and further evaluation.

For all donors, serological results are repeated in triplicate plus NAT as previously described. For sporadic and repeat donors, if test results indicate possible seroconversion, an additional test is conducted using a different serological method. In cases where seroconversion is confirmed through a second sample collection, further confirmatory tests are performed. At this stage, the individual is referred to Health Surveillance services and is considered a patient rather than a donor. Once a donor has a positive result, they will no longer be eligible to donate blood or other blood products.

Following approval by the Research Ethics Committee, contact was established with the *Hemocentro Coordenador de Florianópolis* (Coordinating Florianópolis Hemocenter) to obtain the necessary data for this study. A report was requested from the HemoSis database, generated by the service, and provided in a Microsoft Excel spreadsheet based on the study's eligibility criteria. Blood donors were identified using donation numbers, ensuring anonymity by excluding any nominal identification. The investigators utilised this report for analysis using statistical software.

From the individualised yet anonymised database, the study's variables of interest were reviewed. Sociodemographic data included gender (male, female), age (in complete years), skin colour (white, black, brown, and yellow), education level (illiterate, elementary school, high school, college degree), and marital status (married, single, common‐law marriage, divorced, and widowed). Additionally, data on donor type (first‐time donor, repeat donor, sporadic donor), sample type (first sample—donation; second sample—collected to verify altered results), and donation type (spontaneous—voluntary donation; replacement—donation associated with a specific patient) were examined.

The total number of annual donations during the study period was also collected to calculate the positivity rate, along with the yearly number of HCV‐positive donors, the city of residence of HCV‐positive donors, and serological data (anti‐HCV and NAT results). The hepatitis C positivity rate among blood donors was calculated by dividing the total number of HCV‐positive cases (anti‐HCV and/or NAT) in a given year by the total number of blood donations in the same year, then multiplying the result by 100.000.

The database, received in Microsoft Excel format, was exported to SPSS software, version 21.0 (IBM, Armonk, New York, USA) for statistical analysis. Quantitative variables were described using measures of central tendency and dispersion, while qualitative variables were expressed as absolute frequencies and rates. A 5% level of statistical significance (*p* < 0.05) was adopted. Pearson's chi‐square test and Student's t‐test were used to compare sex and mean ages with both serological and NAT positivity for hepatitis C, respectively.

The temporal trend analysis was conducted using a simple regression model. The regression coefficient was interpreted as the average annual variation in HCV‐positive rate among blood donors, while the model intercept was used to estimate occurrences in the base year. To assess the model's explanatory power, the coefficient of determination (*R* ^2^) was calculated. Microsoft Excel 365 was also used for statistical analysis.

To map the phenomenon under review, Geographic Information System (GIS) software, specifically Quantum GIS (QGIS), along with Microsoft Excel 2016, was used. Data on hepatitis C cases among blood donors were tabulated according to the year of notification and the donor's municipality of residence. Tables were created to summarise the number of cases per year by municipality, adjusted for the total population of each municipality. No data was available on the number of blood donors in each municipality or in the state of Santa Catarina, so for the spatial distribution we used the average population of each municipality over the study period.

The cartographic data used were maps in shape format, in the official Brazilian cartographic system, of the municipalities based on the official data made available by the *Instituto Brasileiro de Geografia e Estatística* (IBGE, Brazilian Institute of Geography and Statistics), as well as the annual population data of the same institute, published in the relevant *Diário Oficial da União* (DOU, Official Gazette).

Using QGIS, a link was made from the municipal codes of the tables of total cases, the population annualised by municipality and, finally, the positivity rate of cases per 100 000 inhabitants was calculated in the same GIS setting, a variable that was mapped thematically, using the representation of classes by Jenks' natural breaks. Jenks' statistical method of natural breaks generates a defined set of thematic classes based on natural groupings according to the types of data that group similar values that maximise the differences between classes; thus, the features are divided into classes where there are relatively large differences in the data values, minimising the sum of variance within each class (clustering method).^17^ This method is suitable for mapping non‐uniformly distributed values, as in the case of our study.

## 3 RESULTS

During the study period, 782 blood donations tested positive for hepatitis C. All donations were positive for the anti‐HCV serological test, and 360 (46.0%) were also positive for NAT. Men had a higher frequency of both serological and NAT positivity for hepatitis C, whereas women had a higher frequency of serological positivity alone (*p* < 0.001).

Individuals with a higher mean age (39.9 ± 10.3 years) were more likely to test positive for both the serological test and NAT, while those with serological positivity alone had a lower mean age (37.7 ± 12 years) (*p* = 0.004). Table 1 presents the sociodemographic characteristics of HCV‐positive donors from 2010 to 2020. The average age of HCV‐positive blood donors was 38.7 years (SD ± 11.3), with ages ranging from 16 to 65 years.

**TABLE 1:** Sociodemographic characteristics among hepatitis C virus (HCV)‐positive blood donors of Public Blood Network of the State of Santa Catarina, 2010–2020 (*n* = 782).

Variable	*n*	%	*n* (%)
HCV‐positive blood donors	782	100.0	NAT positive (*n* = 360)
Gender			
Female	321	41.0	119 (33.1)
Male	461	59.0	241 (66.9)
Age (years)			
16–19	38	4.9	9 (2.5)
20–39	370	47.3	162 (45.0)
40–59	347	44.3	178 (49.4)
≥60	27	3.5	11 (3.1)
Skin colour			
White	743	95.0	345 (95.8)
Black	23	2.9	6 (1.7)
Brown	14	1.8	9 (2.5)
Yellow	2	0.3	–
Education			
Illiterate	6	0.8	2 (0.6)
Elementary school	241	30.9	116 (32.2)
High school	305	39.0	153 (42.5)
College degree	225	28.7	85 (23.6)
Without information	5	0.6	4 (1.1)
Marital status			
Married	324	41.4	150 (41.7)
Single	296	37.9	132 (36.7)
Common law marriage	101	12.9	52 (14.4)
Divorced	45	5.8	18 (5.0)
Widowed	16	2.0	8 (2.2)
Type of donor			
First time donor	367	46.9	222 (61.7)
Repeat donor	9	1.2	1 (0.3)
Sporadic donor	26	3.3	9 (2.5)
Without information	380	48.6	128 (35.6)
Type of sample			
First sample	402	51.4	232 (64.4)
Second sample	380	48.6	128 (35.6)
Reason for donation			
Replacement	295	37.7	166 (46.1)
Spontaneous	107	13.8	66 (18.3)
Without information	380	48.5	128 (35.6)

*Source*: Public Blood Network of the State of Santa Catarina.

The HCV‐positive blood donors in this study were predominantly adults, male, white, married individuals with at least a secondary education. Most of these individuals donated blood for replacement purposes and were first‐time donors.

During the study period, a total of 1 316 605 blood donations were performed. Given that 782 donations tested positive for HCV, the overall positivity rate was 59.4 per 100 000 donations. Table 2 shows the distribution of the positive sample without confirmatory testing in all cases over the study period.

**TABLE 2:** Distribution of rates of hepatitis C virus (HCV) positive in blood samples of Public Blood Network of the State of Santa Catarina, 2010–2020.

Year	Anti‐HCV	Anti‐HCV+NAT	HCV‐positive	Total donations	Rate per 100 000 donations
2010	98	25	123	113 559	108.3
2011	47	68	115	123 690	93.0
2012	60	36	96	121 778	78.8
2013	46	54	100	122 814	81.4
2014	36	42	78	125 831	62.0
2015	32	64	96	126 768	75.7
2016	15	17	32	114 736	27.9
2017	6	14	20	114 765	17.4
2018	21	9	30	120 585	24.9
2019	38	20	58	119 973	48.3
2020	23	11	34	112 106	30.3
Total	422	360	782	1 316 605	59.4

Source: Public Blood Network of the State of Santa Catarina.

**FIGURE 1:**
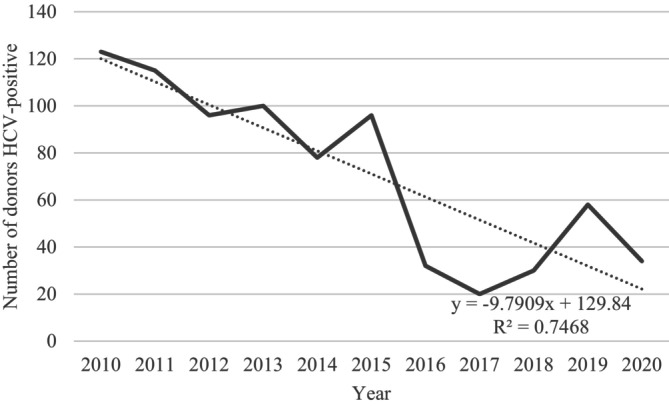
Temporal trend of hepatitis C virus (HCV)‐positive donors in Public Blood Network of the State of Santa Catarina, 2010–2020 (*n* = 782).

A reduction in the positivity rate was observed over the study period, with the highest rates in 2010 and the lowest in 2017 and 2018. Figure 1 displays the temporal distribution of HCV‐positive donors. Over the 10‐year period, there was an average annual decrease of 9.79 HCV‐positive samples, and this reduction was statistically significant (*p* value = 0.0006).

**FIGURE 2:**
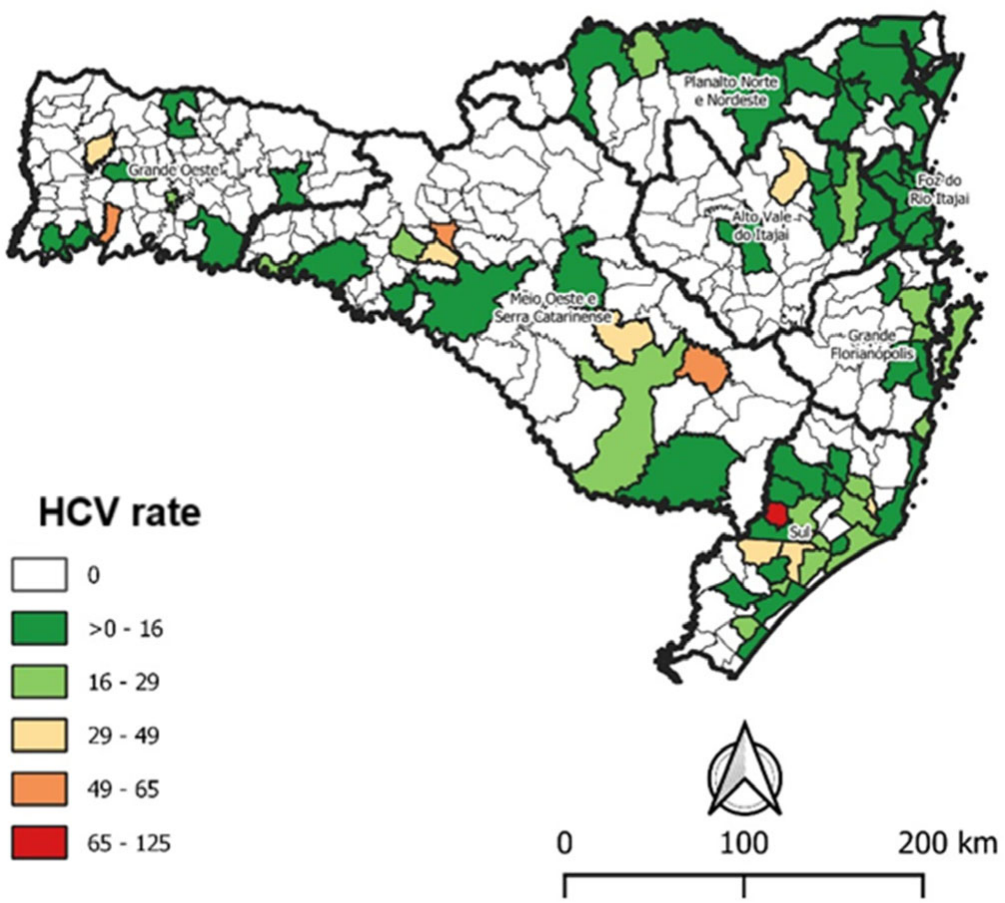
Spatial distribution of rates of hepatitis C virus (HCV)‐positive donors per 100 000 inhabitants in blood samples of Public Blood Network of the State of Santa Catarina, 2010–2020 (*n* = 782).

Figure 2 presents the geographic distribution of HCV‐positive donors by their place of residence in Santa Catarina. The data indicate a predominance of HCV‐positive donors in the southern macro‐region and the coastal region of the state. Additionally, the spatial distribution highlights cities with higher positivity rates in the Alto Vale do Itajaí, Meio Oeste, Serra Catarinense, and Grande Oeste regions.

## 4 DISCUSSION

This study reviewed the positivity rate of hepatitis C in blood donations in Santa Catarina, observing a decreasing trend in positivity and its spatial distribution across the state over a 10‐year period. Among HCV‐positive donors, a single serological marker was predominant. In contrast, a smaller number of blood donors tested positive for both the serological test and NAT simultaneously. It is important to note that a positive anti‐HCV result indicates prior contact with the virus but does not differentiate between past and active infection. On the other hand, a positive NAT result, in addition to serology, confirms an active infection by detecting the presence of viral genetic material in the host.^16^ It is worth noting that these results may be influenced by the fact that NAT was only introduced in blood bank testing starting in 2013.

After infection, HCV reaches hepatocytes and takes control of autophagy—an essential catabolic process in cellular homeostasis—to stimulate its own replication. Through various mechanisms, the virus can temporally alter autophagic flow both directly and indirectly, maximising its replication while suppressing the host's innate immune responses.^18^

Spontaneous clearance of HCV, which occurs without medical intervention, happens at a significantly lower rate compared to hepatitis B virus infection. Evidence suggests that the likelihood of spontaneous clearance decreases in older individuals, men, Black individuals, those coinfected with HIV, and intravenous drug and alcohol users.^19^ In this study, some of these characteristics could not be assessed, representing a data limitation in understanding why a large percentage of donors did not have a positive NAT result. However, older blood donors and men showed a higher frequency of both anti‐HCV and NAT positivity, whereas younger donors and women were more likely to have only a positive serological test for HCV. Additionally, because HCV infection is often asymptomatic, many donors are unaware of their serological status and only discover the infection when donating blood.

The study population was predominantly composed of adult donors, which aligns with the recommended profile for blood component donation. This finding is consistent with studies conducted on blood donors at blood centres in Criciúma, Santa Catarina, and Uberaba, Minas Gerais.^20^, ^21^

Historically, illicit injection drug use, unsafe medical procedures, and contaminated blood transfusions were the primary sources of HCV transmission in the adult population. This is evidenced by the decline in new infections recorded since the 1980s, following improvements in healthcare practices and the adoption of more advanced screening procedures for blood and organ donors.^22^ These new screening measures represent a significant milestone in the history of blood centres in the country. Although hepatitis C prevalence in the general population is associated with older age, individuals over 65 or those with certain medical conditions are not eligible for blood donation, which explains the donor profile observed in this study.

According to data from blood donors in Brazil, a 2020 report showed that more than 3.9 million people donated blood in the country, corresponding to a rate of 14.8 donors per 1000 inhabitants. Of this total, 56% were men, and 67% were over the age of 29. Among the criteria for blood donation ineligibility, 0.37% was due to a positive result for viral hepatitis, although the report did not distinguish between cases of hepatitis B and C.^23^

In this study, hepatitis C cases predominated in males, which is consistent with other studies. In Santa Cruz do Sul, between 2002 and 2015, approximately 67% of hepatitis C cases occurred in males.^24^ Furthermore, in Brazil, 106 637 cases of HCV were reported among men between 1999 and 2015, representing 58.5% of all cases.^25^ A study evaluating the U.S. population also showed higher rates of chronic HCV among men compared to women across all age groups.^26^

The higher prevalence of HCV infection in males can be attributed to various factors, including less consistent condom use during sexual activity and increased exposure to alcohol, injectable drugs, and inhalable substances.^27^ However, risk behaviours for hepatitis C among blood donors could not be assessed in this study. According to the blood bank protocol, after the initial interview, risk factors for bloodborne diseases lead to donor ineligibility, and blood samples are not collected. Therefore, among the HCV‐positive donors in this study, it is not possible to determine the route of infection. It is also important to note that the data related to sex should be interpreted with caution in the present study, since sociodemographic information is not available for all blood donors, with a description provided only for cases that tested positive for HCV.

The temporal trend of the hepatitis C positivity rate in the blood samples assessed showed a linear reduction over the 10 years of the study. This downward trend aligns with the results of other studies covering similar periods, such as those conducted in Europe and China.^28^, ^29^ In this context, it can be suggested that the reported hepatitis C positivity rate will continue to decrease significantly in the long term, as recommended by the World Health Organization in 2016, which outlined a global strategy to eliminate HCV by 2030.^30^ Furthermore, the availability of rapid tests and decentralised diagnosis in Primary Health Care may help prevent infected individuals from donating blood, in addition to promoting the dissemination of preventive measures.^31^

A study conducted in three blood centers located in São Paulo, Belo Horizonte, and Recife, which together account for nearly 8% of all annual blood donations in Brazil, found an HCV prevalence rate of 191 (CI 95% 163–219) per 100 000 donations in 2007.^32^ In Santa Catarina, we observed a rate of 59.4 per 100 000 donations over the study period. It is important to note that a lower rate in this study may be explained by a higher rate of pre‐donation diagnosis, which renders individuals ineligible to donate blood in Brazil—even after successful treatment and cure of hepatitis C. São Paulo and Recife had the highest hepatitis C prevalence rates compared to Belo Horizonte, suggesting a potential association with behavioural risk factors in the transmission chain of hepatitis C.^32^, ^33^

It should be noted that, as part of the blood donation protocol, individuals undergo clinical screening after registration—a non‐serological procedure that excludes people with risk factors for communicable diseases.^16^ Therefore, the positivity rate among blood donors is lower than that in the general population.

According to the geographic distribution of hepatitis C cases in the State of Santa Catarina, the study revealed that the southern macro‐region had the highest number of discarded HCV‐positive blood samples. Another study showed that the city of Criciúma, also located in the southern region of Santa Catarina, exhibited the highest risk of HCV among blood donors.^34^ The spatial distribution map also revealed the predominance of HCV‐positive donors along the entire coast of Santa Catarina. According to a report published by the Board of Epidemiological Surveillance of Santa Catarina, from 2011 to 2021, the findings align with the present study, as the detection rates of hepatitis C in the regions of Foz do Rio Itajaí, Greater Florianópolis, Far South Catarinense, Carbonífera, and Laguna were higher than the state average.^35^

Although the southern and coastal regions of Santa Catarina exhibit a higher positivity rate for hepatitis C, the spatial distribution reveals municipalities with high positivity rates across all macro‐regions of the state, particularly in the regions of Foz do Rio Itajaí, Meio Oeste, Serra Catarinense, and Grande Oeste. An ecological study was conducted to estimate the disability‐adjusted life years (DALY) by adding the number of years of life lost to the number of years lived with disability, based on the hepatitis C viral load in Santa Catarina's general population. According to that study, the highest rates were found in Grande Oeste, with 3024.80 DALY/100000 inhabitants, and the South, with 1212.22 DALY/100000 inhabitants.^36^ The greater disease burden observed in these regions may be partly attributed to the fact that Santa Catarina is a popular tourist destination, with highways that cross the entire state and connect to other states and countries, facilitating HCV transmission.^37^ Additionally, the western region of Santa Catarina is endemic for hepatitis B, which has a similar transmission route to hepatitis C, and cases of co‐infection are often reported, as mentioned previously.^35^ However, it is important to note that the spatial distribution used the total population as the denominator, since there is no information on the number of blood donors in each region. Under Brazilian law, eligible blood donors are healthy individuals aged 16–69, with men permitted to donate up to four times per year and women up to three times per year. For this reason, the rates presented should be interpreted with caution.

Among the limitations of the present study, the use of secondary data is noteworthy, as it limited the inclusion of additional data that could have been valuable for the investigation. Furthermore, many variables had missing data, which should be considered when interpreting the findings. The calculated rates should be interpreted with caution. Since it is not possible to determine the exact number of blood donors in the state of Santa Catarina, the rates were calculated based on the total population of each city in the state (for the spatial distribution), as well as on the total number of blood donations (Table 2). However, there are individuals in the general population who are not eligible to donate blood, which may lead to an underestimation of the calculated rates. Similarly, among blood donations, there may be multiple donations made by the same donor over time. It is important to note that the study population consists of healthy individuals, who may have a lower prevalence of HCV compared to the general population. Despite this, the study provides valuable insight into the disease positivity rate among blood donors, based on a large number of blood donations over a sufficient period. These findings can help improve understanding of the general population in this state and may be generalised to similar populations.

## 5 CONCLUSION

This study provides important data on the behaviour of hepatitis C in Santa Catarina over a period of 10 years. The positivity rate of HCV in blood donations from the Public Blood Network of the State of Santa Catarina appears to be following a downward trend, possibly due to preventive measures and early diagnoses that help prevent unsafe blood donations. The sociodemographic characteristics of the donors remain consistent with those observed in other studies conducted in the region, such as the notable participation of men, adults, and white individuals among those infected. Additionally, although cases of HCV are reported across all macro‐regions, the highest positivity rates are found in the South, Alto Vale do Itajaí, Meio Oeste, Serra Catarinense, and Grande Oeste regions.

In this context, it is important to note that although there was a reduction in HCV positivity among blood donors during the study period, an active surveillance system is essential. Strategies should be developed according to the population profile of each region, with diagnostic efforts extended to all risk groups, alongside the exploration of prevention strategies. The loyalty of blood donors (repeat donors) also contributes to greater safety in the use of blood components.

## AUTHOR CONTRIBUTIONS

Gabriel Tadeu Ossani oversaw the study design, data collection, analysis, and the writing of the manuscript. Parma Gabriel Oscar Cremona performed the statistical analysis and revised the manuscript. Daisson José Trevisol revised the manuscript. Fabiana Schuelter‐Trevisol assisted in the study design, performed the data statistical analysis, revised the manuscript and supervised all stages of the research.

## CONFLICT OF INTEREST STATEMENT

The authors have no competing interests.

## INFORMED CONSENT

Informed consent for patient information to be published in this article was not obtained because it was retrospective, anonymous study with no possibility of direct contact with the blood donors.

## Supporting information


**Figure S1.** Coverage Map of the Blood Network in Santa Catarina.

## Data Availability

The data that support the findings of this study are available from the authors.

## References

[1] Qadir H , Nasir N , Kouser S , et al. Seroprevalence of hepatitis B, hepatitis C, human immunodeficiency virus, syphilis, and malaria among blood donors at tertiary care hospital blood bank. J Pak Med Assoc. 2021;71(3):897‐899. doi:10.47391/JPMA.1344 34057943

[2] WHO . Global Hepatitis Report. World Health Organization; 2017.

[3] World Health Organization . Hepatitis C. Accessed Aug 18. 2022 https://www.who.int/news-room/fact-sheets/detail/hepatitis-c

[4] KDIGO . Clinical practice guideline for the prevention, diagnosis, evaluation, and treatment of hepatitis C in chronic kidney disease. Kidney Int Suppl. 2018;8(3):91‐165. 10.1016/j.kisu.2018.06.001 PMC6336217 30675443

[5] Alqahtani SM , Alsagaby AS , Mir SA , et al. Seroprevalence of viral hepatitis B and C among blood donors in the northern region of Riyadh Province, Saudi Arabia. Health. 2021;9(8):934. doi:10.3390/healthcare9080934 PMC8394786 34442071

[6] Nissen‐Meyer LSH , Seghatchian J . Donor health assessment—when is blood donation safe? Transfus Apher Sci. 2019;58(1):113‐116. doi:10.1016/j.transci.2018.12.016 30630765

[7] Torre P , Aglitti A , Masarone M , Persico M . Viral hepatitis: milestones, unresolved issues, and future goals. World J Gastroenterol. 2021;27(28):4603‐4638. doi:10.3748/wjg.v27.i28.4603 34366625 PMC8326259

[8] Lee MH , Yang HI , Lu SN , et al. Chronic hepatitis C virus infection increases mortality from hepatic and extrahepatic diseases: a community‐based long‐term prospective study. J Infect Dis. 2012;206(4):469‐477. doi:10.1093/infdis/jis385 22811301

[9] Younossi Z , Brown A , Buti M , et al. Impact of eradicating hepatitis C virus on the work productivity of chronic hepatitis C (CH‐C) patients: an economic model from five European countries. J Viral Hepat. 2016;23(3):217‐226. doi:10.1111/jvh.12483 26482680

[10] Di Lorenzo C , Angus AG , Patel AH . Hepatitis C virus evasion mechanisms from neutralizing antibodies. Viruses. 2011;3(11):2280‐2300. doi:10.3390/v3112280 22163345 PMC3230852

[11] Barritt AS , Fried MW . Maximizing opportunities and avoiding mistakes in triple therapy for hepatitis C virus. Gastroenterology. 2012;142(6):1314‐1323. doi:10.1053/j.gastro.2012.02.013 22537438 PMC3683992

[12] Schaefer EA , Chung RT . Anti‐hepatitis C virus drugs in development. Gastroenterology. 2012;142(6):1340‐1350. doi:10.1053/j.gastro.2012.02.015 22537441

[13] World Health Organization . Global Health Sector Strategy on Viral Hepatitis 2016–2021: towards ending viral hepatitis WHO/HIV/2016.06. Accessed July 15. 2022 http://www.who.int/hepatitis/strategy2016-2021/ghss-hep/en/

[14] Ministério da Saúde . do HIV/Aids e das Hepatites Virais. Boletim Epidemiológico Hepatites Virais, 2021. Ministério da Saúde; 2021.

[15] Ministério da Saúde. Secretaria de Vigilância em Saúde . Departamento de Vigilância, Prevenção e Controle das IST, do HIV/Aids e das Hepatites Virais. Protocolo Clínico e Diretrizes Terapêuticas para Hepatite C e Coinfecções; 2019.

[16] Ministério da Saúde. Gabinete do Ministro . Portaria de Consolidação no. 5, de 28 de setembro de 2017. Accessed in Feb 11, 2025. 2017 http://www.portalsinan.saude.gov.br/images/documentos/Legislacoes/Portaria_Consolidacao_5_28_SETEMBRO_2017.pdf

[17] Jenks GF . The data model concept in statistical mapping. Int Yearb Cartogr. 1967;7:186‐190.

[18] Chan ST , Ou JJ . Hepatitis C virus‐induced autophagy and host innate immune response. Viruses. 2017;9(8):224. doi:10.3390/v9080224 28805674 PMC5580481

[19] Rzymski P , Brzdęk M , Dobrowolska K , et al. Like a rolling stone? A review on spontaneous clearance of hepatitis C virus infection. Viruses. 2024;16(9):1386. doi:10.3390/v16091386 39339862 PMC11435954

[20] Silveira L , Schiavon LL , Silva KP , Lopes TB , Zaccaron MR , Narciso‐Schiavon JL . Clinical and epidemiological profile of blood donors with positive serology for viral hepatitis in southern Brazil. Rev Soc Bras Med Trop. 2011;44(3):269‐273. doi:10.1590/s0037-86822011005000028 21552741

[21] Garcia FB , Pereira GA , Martins PR , Moraes‐Souza H . Epidemiological profile of hepatitis C in blood donors at the Uberaba regional blood center. Rev Soc Bras Med Trop. 2009;42(1):1‐4. doi:10.1590/s0037-86822009000100001 19287926

[22] Ansaldi F , Orsi A , Sticchi L , Bruzzone B , Icardi G . Hepatitis C virus in the new era: perspectives in epidemiology, prevention, diagnostics and predictors of response to therapy. World J Gastroenterol. 2014;20(29):9633‐9652. doi:10.3748/wjg.v20.i29.9633 25110404 PMC4123355

[23] Ministério da Saúde. Agência Nacional de Vigilância Sanitária . Boletim de Produção Hemoterápica. Accessed in Feb 11, 2025. 2022 https://www.gov.br/anvisa/pt‐br/assuntos/noticias‐anvisa/2022/anvisa‐divulga‐9o‐boletim‐de‐producao‐hemoterapica

[24] Possuelo LG , Perin D , Breunig PF , et al. Hepatitis C: evaluation of outcomes and georeferencing of cases in Santa Cruz do Sul, Brazil, between 2002 and 2015: a cross‐sectional study. Sao Paulo Med J. 2017;136:109‐115. doi:10.1590/1516-3180.2017.0169180917 29267536 PMC9879543

[25] Ministério da Saúde . Prevenção e Controle das IST, do HIV/Aids e das Hepatites Virais. Boletim Epidemiológico Hepatites Virais. Ministério da Saúde; 2017.

[26] Moore KJ , Gauri A , Koru‐Sengul T . Prevalence and sociodemographic disparities of hepatitis C in baby boomers and the US adult population. J Infect Public Health. 2019;12(1):32‐36. doi:10.1016/j.jiph.2018.08.003 30170837

[27] Stroffolini T , Stroffolini G . Prevalence and modes of transmission of hepatitis C virus infection: a historical worldwide review. Viruses. 2024;16(7):1115. doi:10.3390/v16071115 39066277 PMC11281430

[28] Grubyte S , Urboniene J , Nedzinskiene L , Jancoriene L . The epidemiological patterns of hepatitis C in Lithuania: changes in surveillance from 2005 to 2018. Medicina. 2021;57(10):1120. doi:10.3390/medicina57101120 34684157 PMC8541129

[29] Zhao Q , Jiang S , Li M , et al. Incidence trend and age‐period‐cohort analysis of reported hepatitis C among residents aged 30 to 79 in northeastern China, 2008 to 2017. Medicine. 2020;99(36):e22005. doi:10.1097/MD.0000000000022005 32899048 PMC7478665

[30] Dhiman RK , Grover GS , Premkumar M . Hepatitis C elimination: a public health perspective. Curr Treat Options Gastroenterol. 2019;17(3):367‐377. doi:10.1007/s11938-019-00240-7 31321673

[31] Ministério da Saúde . Biblioteca Virtual em Saúde MS. “Julho Amarelo”: Mês de luta contra as hepatites virais. Accessed Oct 10 2025. 2022 https://bvsms.saude.gov.br/julho-amarelo-mes-de-luta-contra-as-hepatites-virais/#:~:text=A%20campanha%20%E2%80%9CJulho%20Amarelo%E2%80%9D%20foi

[32] de Almeida‐Neto C , Sabino EC , Liu J , et al. Prevalence of serologic markers for hepatitis B and C viruses in Brazilian blood donors and incidence and residual risk of transfusion transmission of hepatitis C virus. Transfusion. 2013;53(4):827‐834. doi:10.1111/j.1537-2995.2012.03840.x 22882510 PMC3499633

[33] Diretoria de Vigilância Epidemiológica . Barriga Verde Informativo Epidemiológico. Accessed September 8, 2022. 2016 https://www.dive.sc.gov.br/phocadownload/boletim-barriga-verde/hepatites-virais/04-Informativo_Hepatites_2016.pdf

[34] Kupek E , Petry A . Changes in the prevalence, incidence and residual risk for HIV and hepatitis C virus in southern Brazilian blood donors since the implementation of NAT screening. Rev Soc Bras Med Trop. 2014;47(4):418‐425. doi:10.1590/0037-8682-0133-2014 25229280

[35] Diretoria de Vigilância Epidemiológica . Barriga Verde Informativo Epidemiológico. Accessed September 10, 2022. 2022 https://www.dive.sc.gov.br/phocadownload/boletim‐barriga‐verde/hepatites‐virais/BBVHepatitesVirais2022.pdf

[36] Traebert J , Fratoni KRBP , Rosa LCDD , Traebert E , Schneinder IJC . The burden of hepatitis C infection in a southern Brazilian state. Rev Soc Bras Med Trop. 2018;51(5):670‐673. doi:10.1590/0037-8682-0098-2017 30304275

[37] Teles SA , Gir E , Martins RMB , Dos Santos Carneiro MA , de Matos MA , Caetano KAA . Emergent predictors of hepatitis C infection among non‐injection drug users. J Infect Public Health. 2018;11(4):526‐529. doi:10.1016/j.jiph.2017.10.008 29097105

