# Supplementary figures and images for: Hepatitis C virus infection in blood donors in Santa Catarina, Brazil, 2010–2020
Source: Transfus Med. 2025 Jul 24;35(5):486–93. doi: 10.1111/tme.70004 (PMC12499645; doi:10.1111/tme.70004)

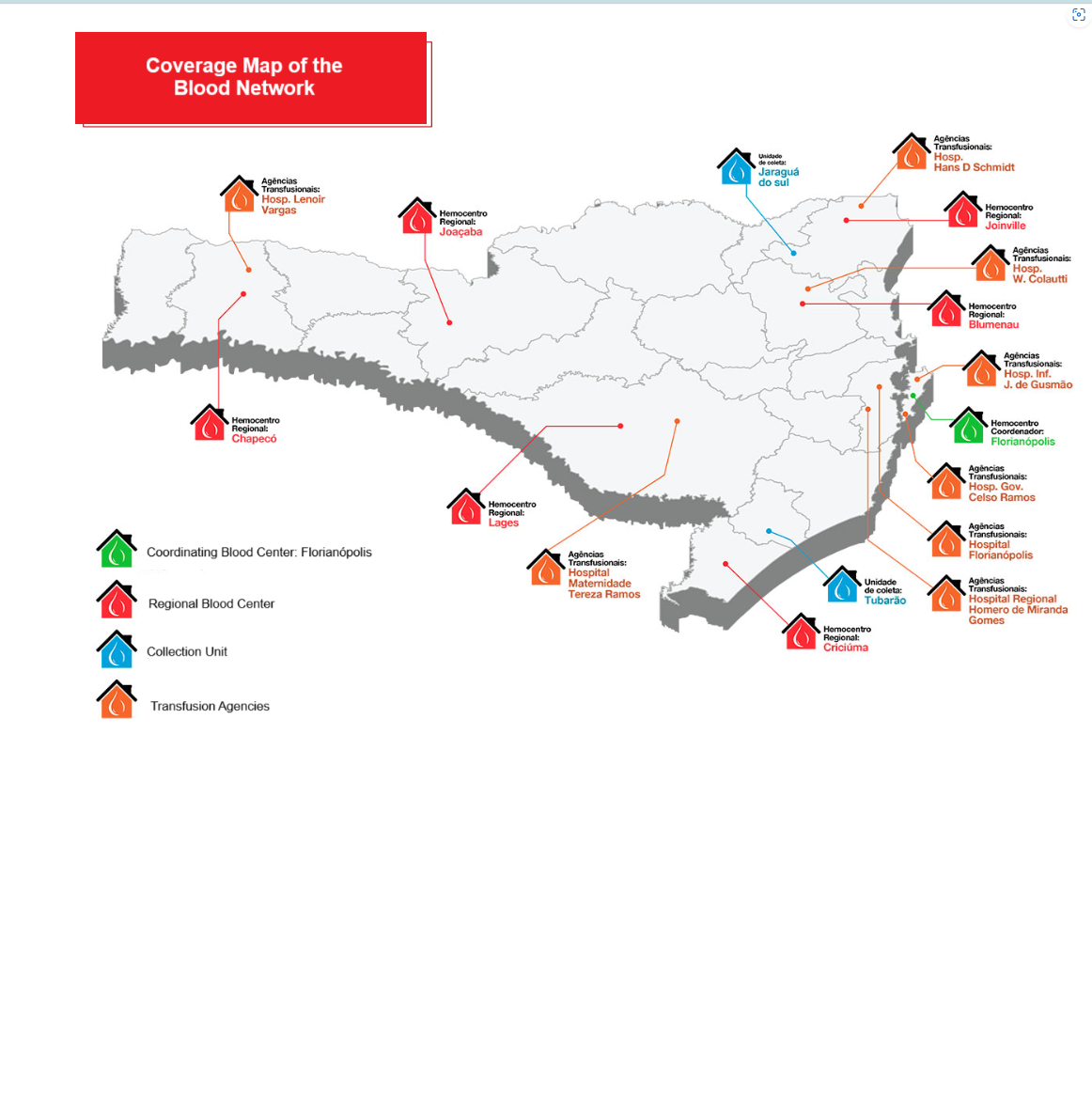

Supplement: Supplementary file 1 — Figure S1. Coverage Map of the Blood Network in Santa Catarina. [file TME-35-486-s001.png]
